# Supplementary material for: Research sites get closer to field camps over time: Informing environmental management through a geospatial analysis of science in the McMurdo Dry Valleys, Antarctica
Source: PLoS One. 2021 Nov 4;16(11):e0257950. doi: 10.1371/journal.pone.0257950 (PMC8568199; doi:10.1371/journal.pone.0257950)
Supplement: S1 File — This describes the process and parameters used to create the heat maps (Fig 4 and S1 Video). (DOCX) [file pone.0257950.s001.docx]

**Protocol A.** **Kernel density estimation (KDE) parameterization**

To parameterize the KDE, we bound the analysis to the study site minimum bounding polygon and set the output grid cell size to 1.0 km. We set the ‘population’ field to the number of publications at each site. We set the ‘method’ to PLANAR, and ‘Output Values’ to “Expected_Counts”. Adapting the approach of Blackburn et al. (2014), we set the search radius (or ‘bandwidth’) to the value determined by the optimal bandwidth function, defined by (Fotheringham et al. (2000):

$$h_{opt}= \left[ \frac{2}{3n} \right]^{\left( \frac{1}{4} \right)}\sigma,$$

where *n* is the sample size (number of sites studied) and *σ* is the standard distance of the site locations. We calculated all standard distances at 1 standard deviation. For a cumulative summary of publication density, we conducted a KDE using the full study site data for all years (h_opt_ = 4.971 km) (Fig. 4 in main manuscript). To compare publication density over time, we computed h_opt_ for each year and averaged the values (Nelson and Boots 2008; Blackburn et al. 2014), and then used the mean (h_opt_ =13.168 km) as the search radius in a KDE for each year (S4 Video). We omitted data from 1914 and 1916 from this analysis because they contained too few features to compute standard distance.

References:

1. Blackburn JK, Hadfield TL, Curtis AJ, Hugh-Jones ME. Spatial and Temporal Patterns of Anthrax in White-Tailed Deer, Odocoileus virginianus, and Hematophagous Flies in West Texas during the Summertime Anthrax Risk Period. Annals of the Association of American Geographers. 2014;104: 939–958. doi:10.1080/00045608.2014.914834

2. Fotheringham AS, Brunsdon C, Charlton M. Quantitative Geography: Perspectives on Spatial Data Analysis. SAGE Publications; 2000. Available: https://books.google.ca/books?id=QuEIadpO1oEC

3. Nelson TA, Boots B. Detecting spatial hot spots in landscape ecology. Ecography. 2008;31: 556–566. doi:10.1111/j.0906-7590.2008.05548.x
