# Supplementary material for: Research sites get closer to field camps over time: Informing environmental management through a geospatial analysis of science in the McMurdo Dry Valleys, Antarctica
Source: PLoS One. 2021 Nov 4;16(11):e0257950. doi: 10.1371/journal.pone.0257950 (PMC8568199; doi:10.1371/journal.pone.0257950)
Supplement: S2 File — Details on the review and screening process for bibliographic references. (DOCX) [file pone.0257950.s002.docx]

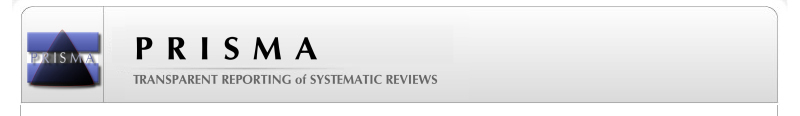
**PRISMA Flow Diagram**

284 unique study sites with reliable geolocation data

(156 from GNIS-PGC)

(128 from MDV-LTER)

Studies included in bibliometric and geospatial analyses
(n = 1486)

(Sampling in MDV = ‘yes’)

Additional records identified from New Zealand paperbound bibliographies (Vol. 1–3)
(n = 1569)

Records excluded:

- Additional duplicates

(n = 22)

Full-text articles assessed for eligibility
(n = 2720)

Full-text articles excluded:

- Full text unavailable

- Non-English

- Insufficient location info

- Review or synthesis

- Remote sensing survey

(n = 1234)

Records screened
(n = 2742)

Records after duplicates (n = 84) removed
(n = 2742)

## Identification

## Eligibility

## Included

## Screening

Records identified through Web of Science database query
(n = 1257)
