# Supplementary material for: Research sites get closer to field camps over time: Informing environmental management through a geospatial analysis of science in the McMurdo Dry Valleys, Antarctica
Source: PLoS One. 2021 Nov 4;16(11):e0257950. doi: 10.1371/journal.pone.0257950 (PMC8568199; doi:10.1371/journal.pone.0257950)
Supplement: S1 Dataset — Bibliographic data; camp distance frequency tables and statistical tests for the proximity analysis (Figs 3 and 5); Table A-Location analysis/quartile counts and Table B-Camp rankings by publication (Fig 6). (ZIP) [file pone.0257950.s003.zip › S2_Dataset/Table_A-location_analysis_quartile_counts.docx]

**Table A. Location analysis quartile counts**

|  | **Asgard** | **Brownworth** | **Bull Pass** | **F6** | **Fryxell SSSI** | **Bonney Old** | **Bonney New** | **Fryxell Camp** | **Lake Hoare** | **Lower Wright** | **Marble Point** | **Meserve** | **Miers** | **New Harbor** | **Vanda New** | **Vanda Old** |
| --- | --- | --- | --- | --- | --- | --- | --- | --- | --- | --- | --- | --- | --- | --- | --- | --- |
| **Nationality** | NZ | NZ | US | US | NZ | US | US | US | US | NZ | US | US | NZ | US | NZ | NZ |
| **Duration** | 26 | 16 | 30 | 22 | 17 | 16 | 27 | 29 | 37 | 32 | 59 | 14 | 17 | 34 | 22 | 26 |
| **Quartile duration*** | 7 | 4 | 8 | 6 | 4 | 4 | 7 | 7 | 9 | 8 | 15 | 4 | 4 | 9 | 6 | 7 |
| **Start Year**** | 1969 | 1969 | 1987 | 1995 | (1978)  1979 | 1962 | 1990 | (1988)  1989 | (1980)  1981 | 1985 | 1958 | 1969 | (1979) 1980 | 1983 | 1995 | 1969 |
| **Final Year**** | (1994)  1996 | 1984 | [2016]  +2 | [2016]  +2 | 1994 | 1977 | [2016] +1 | [2016] | [2016] | [2016] | [2016] +1 | (1982)  1984 | 1995 | [2016] +2 | [2016] +2 | (1994)  1996 |
| **Total Pubs** | 746 | 197 | 826 | 1073 | 679 | 412 | 976 | 1106 | 1378 | 461 | 182 | 588 | 59 | 1053 | 779 | 774 |
| **Total Pubs During Hut** | 549 | 134 | 283 | 698 | 325 | 254 | 611 | 676 | 990 | 239 | 181 | 403 | 31 | 740 | 207 | 572 |
| **Percent Pubs**  **During Hut %** | 74 | 68 | 34 | 65 | 48 | 62 | 63 | 61 | 72 | 52 | 99 | 69 | 53 | 70 | 27 | 74 |
| **Total Pubs <5** | 12 | 6 | 38 | 421 | 267 | 130 | 316 | 396 | 394 | 20 | 56 | 79 | 31 | 99 | 245 | 273 |
| **Total Pubs <5**  **During Hut** | 12 | 5 | 16 | 282 | 145 | 88 | 183 | 297 | 329 | 15 | 56 | 61 | 17 | 19 | 59 | 198 |
| **Percent Pubs <5 During Hut** | 100 | 83 | 42 | 67 | 54 | 68 | 58 | 75 | 84 | 75 | 100 | 77 | 55 | 19 | 24 | 73 |
| **Q1 <5 km Increase** | 0 to 2 | 0 to 0 | 6 to 8 | 27 to 28 | 30 to 50 | 3 to 17 | 15 to 25 | 31 to 27 | 48 to 49 | 5 to 1 | 0 to 27 | 7 to 23 | 4 to 5 | 78 to 4 | 26 to 10 | 41 to 66 |
| **Q1 <5 km Total Increase** | 2 | 0 | 2 | 1 | 20 | 14 | 10 | -4 | 1 | -4 | 27 | 16 | 1 | -74 | -16 | 25 |
| **Q1+Q2 <5 km Increase** | 0 to 8 | 0 to 0 | 19 to 11 | 48 to 103 | 42 to 88 | 3 to 37 | 61 to 62 | 65 to 83 | 62 to 88 | 5 to 2 | 0 to 32 | 10 to 38 | 7 to 13 | 80 to 5 | 59 to 23 | 43 to 66 |
| **Q1+Q2 <5 km Total Increase** | 8 | 0 | -8 | 55 | 46 | 34 | 1 | 18 | 26 | -3 | 32 | 28 | 6 | -75 | -36 | 94 |
| **Q4 <5 km Decrease** | 0 to 0 | 3 to 0 | N/A | N/A | 30 to 21 | 36 to 19 | N/A | N/A | N/A | N/A | N/A | 10 to 4 | 2 to 6 | N/A | N/A | 22 to 13 |
| **Q4 <5 km Total Decrease** | 0 | 3 | N/A | N/A | 9 | 17 | N/A | N/A | N/A | N/A | N/A | 6 | -4 | N/A | N/A | 9 |
| **Q3+Q4 <5 km Decrease** | 4 to 0 | 5 to 1 | N/A | N/A | 57 to 80 | 51 to 39 | N/A | N/A | N/A | N/A | N/A | 23 to 8 | 4 to 7 | N/A | N/A | 61 to 32 |
| **Q3+Q4 <5 km Decrease** | 4 | 4 | N/A | N/A | -23 | 12 | N/A | N/A | N/A | N/A | N/A | 15 | -3 | N/A | N/A | 29 |

* **Decision rules for quartile analysis:**

- Include both the year the camp was established and the year it was removed in counting of study sites
- If the duration of a camp is an odd number of years, omit the first year
- If possible, divide by four
  - If one over divisible by four: omit first year
  - If one below divisible by four: add the year after*
  - If two over divisible by four: add two years after*
    - *Does not work for current field camps. In this case: add the average of the final quartile

** Actual dates are outside brackets. Brackets indicate dates used to calculate quartiles. Square brackets indicate hut is still in existence in 2016/2017.

**Individual Hut Data by Quartile**

Asgard

|  | 2^nd^ Q Before  1955  1961 | 1^st^ Q Before  1962  1968 | **1^st^ Quartile**  **1969- 1975** | **2^nd^ Quartile**  **1976-1982** | **3^rd^ Quartile**  **1983-**  **1989** | **4^th^ Quartile**  **1990- 1996** | 1^st^ Q After  1997-  2003 | 2^nd^ Q After  2004-  2010 |
| --- | --- | --- | --- | --- | --- | --- | --- | --- |
| <5 | 0 | 0 | 2 | 6 | 4 | 0 | 0 | 0 |
| 5-20 | 4 | 74 | 161 | 190 | 121 | 65 | 34 | 85 |

Brownworth

|  | 2^nd^ Q Before  1961-  1964 | 1^st^ Q Before  1965-  1968 | **1^st^ Quartile**  **1969-1972** | **2^nd^ Quartile**  **1973-1976** | **3^rd^ Quartile**  **1977- 1980** | **4^th^ Quartile**  **1981-**  **1984** | 1^st^ Q After  1985- 1988 | 2^nd^ Q After  1989-  1992 |
| --- | --- | --- | --- | --- | --- | --- | --- | --- |
| <5 | 0 | 0 | 0 | 0 | 2 | 3 | 0 | 1 |
| 5-20 | 14 | 23 | 40 | 30 | 30 | 29 | 16 | 9 |

Bull Pass

|  | 4^th^ Q Before  1955-  1962 | 3^rd^ Q  Before  1963-  1970 | 2^nd^ Q Before  1971-  1978 | 1^st^ Q Before  1979-  **1986** | **1^st^ Quartile**  **1987-**  **1994** | **2^nd^ Quartile**  **1995-**  **2002** | **3^rd^ Quartile**  **2003-**  **2010** | **4^th^ Quartile**  **2011-**  **2016 +2** |
| --- | --- | --- | --- | --- | --- | --- | --- | --- |
| <5 | 0 | 3 | 13 | 6 | 8 | 3 | 3 | 2 |
| 5-20 | 23 | 82 | 265 | 151 | 86 | 28 | 89 | 64 |

F6 Hut

|  | 4^th^ Q Before  1971-  1976 | 3^rd^ Q  Before  1977-  1982 | 2^nd^ Q Before  1983-1988 | 1^st^ Q Before  1989- 1994 | **1^st^ Quartile**  **1995-2000** | **2^nd^ Quartile**  **2001-2006** | **3^rd^ Quartile**  **2007-2012** | **4^th^ Quartile**  **2013**  **2018** |
| --- | --- | --- | --- | --- | --- | --- | --- | --- |
| <5 | 29 | 62 | 21 | 27 | 28 | 75 | 65 | 114 |
| 5-20 | 67 | 106 | 38 | 25 | 37 | 132 | 82 | 165 |

Fryxell SSSI

|  | 2^nd^ Q Before  1970-  1973 | 1^st^ Q Before  1974-1977 | **1^st^ Quartile**  **1978-**  **1981** | **2^nd^ Quartile**  **1982-**  **1985** | **3^rd^ Quartile**  **1986-**  **1989** | **4^th^ Quartile**  **1990-1993** | 1^st^ Q After  1994-1997 | 2^nd^ Q After  1998-2001 |
| --- | --- | --- | --- | --- | --- | --- | --- | --- |
| <5 | 12 | 30 | 50 | 38 | 27 | 30 | 21 | 59 |
| 5-20 | 72 | 100 | 95 | 37 | 12 | 36 | 9 | 51 |

Lake Bonney (Old)

|  | 2^nd^ Q Before  1954-1957 | 1^st^ Q Before  1958-1961 | **1^st^ Quartile**  **1962-**  **1965** | **2^nd^ Quartile**  **1966-1969** | **3^rd^ Quartile**  **1970-1973** | **4^th^ Quartile**  **1974-1977** | 1^st^ Q After  1978-1981 | 2^nd^ Q After  1982-1985 |
| --- | --- | --- | --- | --- | --- | --- | --- | --- |
| <5 | 0 | 3 | 17 | 20 | 15 | 36 | 19 | 20 |
| 5-20 | 0 | 4 | 20 | 38 | 50 | 58 | 64 | 48 |

Lake Bonney (New)

|  | 4^th^ Q Before  1962-1968 | 3^rd^ Q  Before  1969-1975 | 2^nd^ Q Before  1976-1982 | 1^st^ Q Before  1983-1989 | **1^st^ Quartile**  **1990-1996** | **2^nd^ Quartile**  **1997-2003** | **3^rd^ Quartile**  **2004-2010** | **4^th^ Quartile**  **2011-2016 (+1)** |
| --- | --- | --- | --- | --- | --- | --- | --- | --- |
| <5 | 37 | 35 | 46 | 15 | 25 | 37 | 53 | 68 |
| 5-20 | 41 | 63 | 89 | 39 | 42 | 89 | 148 | 149 |

Lake Fryxell

|  | 4^th^ Q Before  1961-1967 | 3^rd^ Q  Before  1968-1974 | 2^nd^ Q Before  1975-1981 | 1^st^ Q Before  1982-1988 | **1^st^ Quartile**  **1989-1995** | **2^nd^ Quartile**  **1996-2002** | **3^rd^ Quartile**  **2003-2009** | **4^th^ Quartile**  **2010-2016** |
| --- | --- | --- | --- | --- | --- | --- | --- | --- |
| <5 | 15 | 19 | 34 | 31 | 27 | 56 | 95 | 119 |
| 5-20 | 34 | 71 | 164 | 52 | 32 | 70 | 136 | 141 |

Lake Hoare

|  | 4^th^ Q Before  1945-1953 | 3^rd^ Q  Before  1954-1962 | 2^nd^ Q Before  1963-1971 | 1^st^ Q Before  1972-1980 | **1^st^ Quartile**  **1981-1989** | **2^nd^ Quartile**  **1990-1998** | **3^rd^ Quartile**  **1999-2007** | **4^th^ Quartile**  **2008-2016** |
| --- | --- | --- | --- | --- | --- | --- | --- | --- |
| <5 | 0 | 3 | 14 | 48 | 49 | 39 | 110 | 131 |
| 5-20 | 0 | 17 | 95 | 211 | 118 | 82 | 213 | 248 |

Lower Wright

|  | 4^th^ Q Before  1953-  1960 | 3^rd^ Q  Before  1961-1968 | 2^nd^ Q Before  **1969-1976** | 1^st^ Q Before  **1977-1984** | **1^st^ Quartile**  **1985-1992** | **2^nd^ Quartile**  **1993-2000** | **3^rd^ Quartile**  **2001-2008** | **4^th^ Quartile**  **2009-2016** |
| --- | --- | --- | --- | --- | --- | --- | --- | --- |
| <5 | 0 | 0 | 0 | 5 | 1 | 1 | 5 | 8 |
| 5-20 | 6 | 49 | 89 | 73 | 29 | 17 | 94 | 82 |

Marble Point

|  | 4^th^ Q Before  1898-1912 | 3^rd^ Q  Before  1913-1927 | 2^nd^ Q Before  **1928-1942** | 1^st^ Q Before  **1943-1957** | **1^st^ Quartile**  **1958-1972** | **2^nd^ Quartile**  **1973-1987** | **3^rd^ Quartile**  1988-2002 | **4^th^ Quartile**  2003-2016 (+1) |
| --- | --- | --- | --- | --- | --- | --- | --- | --- |
| <5 | 0 | 0 | 0 | 0 | 27 | 5 | 6 | 18 |
| 5-20 | 0 | 1 | 0 | 0 | 22 | 64 | 6 | 33 |

Meserve

|  | 2^nd^ Q Before  1961-1964 | 1^st^ Q Before  1965-1968 | **1^st^ Quartile**  **1969-1972** | **2^nd^ Quartile**  **1973-1976** | **3^rd^ Quartile**  **1977-1980** | **4^th^ Quartile**  **1981-1984** | 1^st^ Q After  1985  1988 | 2^nd^ Q After  1989-1992 |
| --- | --- | --- | --- | --- | --- | --- | --- | --- |
| <5 | 3 | 7 | 23 | 15 | 13 | 10 | 4 | 4 |
| 5-20 | 34 | 47 | 60 | 87 | 91 | 104 | 47 | 39 |

Miers

|  | 2^nd^ Q Before  1972-1975 | 1^st^ Q Before  1976  1979 | **1^st^ Quartile**  **1980-1983** | **2^nd^ Quartile**  **1984-1987** | **3^rd^ Quartile**  **1988-1991** | **4^th^ Quartile**  **1992-1995** | 1^st^ Q After  1996-1999 | 2^nd^ Q After  2000-2003 |
| --- | --- | --- | --- | --- | --- | --- | --- | --- |
| <5 | 3 | 4 | 5 | 8 | 2 | 2 | 6 | 1 |
| 5-20 | 8 | 5 | 6 | 6 | 1 | 1 | 1 | 0 |

New Harbour

|  | 4^th^ Q Before  1947-1955 | 3^rd^ Q  Before  1956-1964 | 2^nd^ Q Before  **1965-1973** | 1^st^ Q Before  **1974-1982** | **1^st^ Quartile**  **1983-1991** | **2^nd^ Quartile**  **1992-2000** | **3^rd^ Quartile**  **2001-2009** | **4^th^ Quartile**  **2010-2016 (+2)** |
| --- | --- | --- | --- | --- | --- | --- | --- | --- |
| <5 | 0 | 0 | 2 | 78 | 4 | 1 | 5 | 9 |
| 5-20 | 0 | 24 | 76 | 133 | 77 | 88 | 261 | 295 |

Vanda (New)

|  | 4^th^ Q Before  1971-1976 | 3^rd^ Q  Before  1977-1982 | 2^nd^ Q Before  **1983-1988** | 1^st^ Q Before  **1989-1994** | **1^st^ Quartile**  **1995-2000** | **2^nd^ Quartile**  **2001-2006** | **3^rd^ Quartile**  **2007-2012** | **4^th^ Quartile**  **2013-2016 (+2)** |
| --- | --- | --- | --- | --- | --- | --- | --- | --- |
| <5 | 61 | 66 | 33 | 26 | 10 | 13 | 12 | 24 |
| 5-20 | 132 | 122 | 86 | 46 | 16 | 22 | 63 | 47 |

Vanda (Old)

|  | 2^nd^ Q Before  1955-1961 | 1^st^ Q Before  1962-1968 | **1^st^ Quartile**  **1969-1975** | **2^nd^ Quartile**  **1976-1982** | **3^rd^ Quartile**  **1983-1989** | **4^th^ Quartile**  **1990-1996** | 1^st^ Q After  1997-  2003 | 2^nd^ Q After  2004-2010 |
| --- | --- | --- | --- | --- | --- | --- | --- | --- |
| <5 | 2 | 41 | 66 | 71 | 39 | 22 | 13 | 19 |
| 5-20 | 3 | 38 | 127 | 132 | 77 | 38 | 19 | 67 |
