# Supplementary material for: Research sites get closer to field camps over time: Informing environmental management through a geospatial analysis of science in the McMurdo Dry Valleys, Antarctica
Source: PLoS One. 2021 Nov 4;16(11):e0257950. doi: 10.1371/journal.pone.0257950 (PMC8568199; doi:10.1371/journal.pone.0257950)
Supplement: S1 Dataset — Bibliographic data; camp distance frequency tables and statistical tests for the proximity analysis (Figs 3 and 5); Table A-Location analysis/quartile counts and Table B-Camp rankings by publication (Fig 6). (ZIP) [file pone.0257950.s003.zip › S2_Dataset/Table_B-camps-rankings-by-publications.docx]

**Table B. Camp rankings by publications**

Rankings of field camps by total publications and total publications while field camp is in place within a 20 km radius and within a 5 km radius. Note: some publications are counted multiple times due to overlapping buffer areas. These data are visualized in Fig. 6 in the text.

|  | **Total publications**  **< 20 km** | | **Publications < 20 km during camp lifespan** | | **Total Publications**  **< 5 km** | | **Publications < 5 km during camp lifespan** | |
| --- | --- | --- | --- | --- | --- | --- | --- | --- |
| **Rank** | **Pubs.** | **Field Camp** | **Pubs.** | **Field Camp** | **Pubs.** | **Field Camp** | **Pubs.** | **Field Camp** |
| **1** | 1378 | Lake Hoare Camp | 990 | Lake Hoare Camp | 421 | F6 Camp | 329 | Lake Hoare Camp |
| **2** | 1106 | Lake Fryxell Camp | 740 | New Harbor Camp | 396 | Lake Fryxell Camp | 297 | Lake Fryxell Camp |
| **3** | 1073 | F6 Camp | 698 | F6 Camp | 394 | Lake Hoare Camp | 282 | F6 Camp |
| **4** | 1053 | New Harbor Camp | 676 | Lake Fryxell Camp | 316 | Lake Bonney Camp (New) | 198 | Vanda Station (Old) |
| **5** | 976 | Lake Bonney Camp (New) | 611 | Lake Bonney Camp (New) | 273 | Vanda Station (Old) | 183 | Lake Bonney Camp (New) |
| **6** | 826 | Bull Pass Huts | 572 | Vanda Station Old | 267 | Fryxell SSSI Hut | 145 | Fryxell SSSI Hut |
| **7** | 779 | Vanda Station (New) | 549 | Asgard Hut | 245 | Vanda Station (New) | 88 | Lake Bonney Camp (Old) |
| **8** | 774 | Vanda Station (Old) | 403 | Meserve Hut | 130 | Lake Bonney Camp (Old) | 61 | Meserve Hut |
| **9** | 746 | Asgard Hut | 325 | Fryxell SSSI Hut | 99 | New Harbor Camp | 59 | Vanda Station (New) |
| **10** | 679 | Fryxell SSSI Hut | 283 | Bull Pass Huts | 79 | Meserve Hut | 56 | Marble Point Refueling Station |
| **11** | 588 | Meserve Hut | 254 | Lake Bonney Camp (Old) | 56 | Marble Point Refueling Station | 19 | New Harbor Camp |
| **12** | 461 | Lower Wright Hut | 239 | Lower Wright Hut | 38 | Bull Pass Huts | 17 | Miers Hut |
| **13** | 412 | Lake Bonney Camp (Old) | 207 | Vanda Station (New) | 31 | Miers Hut | 16 | Bull Pass Huts |
| **14** | 197 | Brownworth Hut | 181 | Marble Point Refueling Station | 20 | Lower Wright Hut | 15 | Lower Wright Hut |
| **15** | 182 | Marble Point Refueling Station | 134 | Brownworth Hut | 12 | Asgard Hut | 12 | Asgard Hut |
| **16** | 59 | Miers Hut | 31 | Miers Hut | 6 | Brownworth Hut | 5 | Brownworth Hut |

**Total Publications:** 11,289

**Pubs. While Camp in Place:** 6,893

**Total Publications < 5 km:** 2,783

**Pubs. While Camp in place:** 1,782
